# Supplementary material for: Q-Cell Glioblastoma Resource: Proteomics Analysis Reveals Unique Cell-States Are Maintained in 3D Culture
Source: Cells. 2020 Jan 21;9(2):267. doi: 10.3390/cells9020267 (PMC7072469; doi:10.3390/cells9020267)
Supplement: Supplementary file 1 [file cells-09-00267-s001.zip › sup/Dsouza et al 2020 Supplementary Materials-revision.docx]

**Supplementary Materials**

**Figure S1:** Cell-state scores of thirteen Q-cell lines. Distribution of Z-scored ssGSEA scores for MES-like (a), NPC-like (b), AC-like (c) and OPC-like (d) cell-states according to Suva et al. Abbreviations MES-like: mesenchymal-like, NPC-like: neural progenitor cell-like, AC-like: astrocyte-like, OPC-like: oligodendrocyte progenitor cell-like.

**Figure S2:** PCA plot showing the distribution of the cell lines across the four cell-states from the triplicate experiments.

| Pearson's Co-efficient of correlation (protein vs mRNA) | |
| --- | --- |
| BAH1 | 0.41 |
| FPW1 | 0.32 |
| HW1 | 0.49 |
| JK2 | 0.28 |
| MMK1 | 0.48 |
| MN1 | 0.44 |
| PB1 | 0.78 |
| RKI1 | 0.49 |
| RN1 | 0.35 |
| SB2 | N/A |
| SB2b | 0.55 |
| SJH1 | 0.18 |
| WK1 | 0.33 |

**Figure S2:** Pearson’s co-efficient of correlation comparing mRNA and protein expression from transcriptomics and proteomics respectively.

a

**Figure S4:** Functional analysis of the expressed transcriptome. Heatmap of genes (z-scored counts) belonging to the KEGG cancer pathway and GBM associated genes. Dendrogram depicts the similarity in protein profiles among the 12 cell lines. Colour legend: Blue -low expression and yellow - high expression
